# Single-Lead Fetal ECG Extraction Based on a Parallel Marginalized Particle Filter

**DOI:** 10.3390/s17061456

**Published:** 2017-06-21

**Authors:** Zhidong Zhao, Huiling Tong, Yanjun Deng, Wen Xu, Yefei Zhang, Haihui Ye

**Affiliations:** 1 College of Electronics and Information, Hangzhou Dianzi University; Hangdian Smart City Research Center of Zhejiang Province, Hangzhou 310018, China; 2 School of Communication Engineering, Hangzhou Dianzi University, Hangzhou 310018, China; rachelthl07@gmail.com (H.T.); dengyanjun79@gmail.com (Y.D.); xwen0505@gmail.com (W.X.); Valora.Zhang@gmail.com (Y.Z.); 3 Women’s Hospital School of Medicine Zhejiang University, Hangzhou 310006, China; yhaihui0505@gmail.com

**Keywords:** Fetal ECG, marginalized particle filter, ECG dynamical model, extended Kalman filter

## Abstract

This paper presents a novel method for extracting the fetal ECG (FECG) from a single-lead abdominal signal. A dynamical model for a modified abdominal signal is proposed, in which both the maternal ECG (MECG) and the FECG are modeled, and then a parallel marginalized particle filter (par-MPF) is used for tracking the abdominal signal. Finally, the FECG and MECG are simultaneously separated. Several experiments are conducted using both simulated and clinical signals. The results indicate that the method proposed in this paper effectively extracts the FECG and outperforms other Bayesian filtering algorithms.

## 1. Introduction

Fetal ECG (FECG) reflects changes in fetal heart activity and is used as a primary method for evaluating fetal heart status. However, there is no uniform, effective, non-invasive FECG signal elicitation and processing technique, partly because the FECG obtained with an abdominal sensor has a low signal-to-noise ratio and is contaminated by strong interference [1], including from the maternal ECG (MECG), fetal brain activity, and movement artifacts. An additional problem is the lack of a standard FECG database as well as a lack of knowledge of fetal cardiac function. Hence, the analysis of FECG is still in its infancy.

Currently, sophisticated and precise biomedical amplifiers can be used to obtain ECG signals. Accordingly, extracting the FECG from these recordings has gained considerable attention [2]. A number of effective algorithms have been proposed for FECG extraction, and most of these techniques require a multi-lead input. However, the number of leads affects the performance of the algorithm. Extracting the FECG with a single lead would both be more convenient and have a lower cost. For this purpose, many methods have been examined.

Adaptive filtering [3,4] is a simple and fast approach for FECG extraction, and conventional adaptive filtering is based on training an adaptive filter to remove the MECG using a maternal reference signal. However, the fetal ECG extracted using this algorithm is still influenced by the maternal ECG and other disturbances. An adaptive comb filter [5] is used for the FECG estimation from the abdominal signal, and the filter can adjust itself to the temporal variations in the fundamental frequency, which makes it qualified for the estimation of a quasi-periodic component from the abdominal signal. An important advantage of the adaptive comb filter over conventional adaptive filters is its ability to separate the MECG and FECG with a temporal overlap; additionally, it requires only one sensor.

Wavelet-based [6,7,8] methods have shown promising results. The properties of the wavelet transformation allow the extraction of the ECG waveforms from noise and artifact cancellation. These methods require the proper selection of the mother wavelet and scale to identify the frequency components in the signal. However, the selection of the mother wavelet is extremely important for the FECG and MECG delineation. An optimal wavelet should consider the edge variation of the ECG waveforms, which may be adapted to the signal morphology because a different mother wavelet can provide different results. In the case of waveform reconstruction, EMD [9] for respiratory signal evaluation shows better performance.

Extended Kalman filtering (EKF)/extended Kalman smoother (EKS) [10,11,12] has also been applied to extract FECG, which is a synthetic dynamic ECG model within an EKF framework that has been extended to jointly model several ECGs to extract the desired ECGs from an abdominal recording of maternal and fetal ECGs and noise. The results show that model can obtain the FECG and has a high signal-to-noise ratio. However, these methods should approximate the posterior density with a Gaussian density. If the assumption does not hold, the prevailing Bayesian filtering methods that are based on the Gaussian model [13] may fail to address the non-Gaussian and nonlinear FECG signals. Thus, another technique, referred to as MPF, is proposed in this paper.

In this paper, a modified abdominal signal dynamical model containing MECG and FECG is proposed. Both of the new ECG dynamical models have 17 state variables that allow the artificial ECG to adapt to normal or abnormal morphologies. A parallel marginalized particle filter is then used for tracking the abdominal signal. Finally, the FECG and MECG are separated at the same time from an abdominal signal. To evaluate the proposed Bayesian method, both simulated signal experiments and clinical signal experiments were conducted in this paper. Additionally, the performance of the filter was compared with several other Bayesian algorithms.

The rest of this paper is organized as follows: in Section 2, the previously developed synthetic ECG dynamical models are described. The proposed methodology is fully described in Section 3. In Section 4, a description of the performance indices that have been used to estimate the performance of the proposed methods is presented. The results and discussion of the different filtering methods are presented in Section 5; finally, in Section 6, some of the main conclusions are noted.

## 2. ECG Dynamical Model

The ECG dynamical equations, which describe the states used in the KF, were proposed by McSharry in 2003 [14]. The dynamical model is used for generating a synthetic ECG with Cartesian coordinates and consists of three nonlinear dynamical state equations, as follows:

(1) $$\{\begin{array}{c} \dot{x}=\alpha x-\omega y\\ \dot{y}=\alpha y+\omega x\\ \dot{z}=-\sum_{i\in \{Q,P,R,S,T\}}a_{i}\Delta \theta_{i}\exp(-\frac{\Delta \theta_{i}}{2b_{i}})-(z-z_{0}) \end{array}$$

where $\alpha =1-\sqrt{x^{2}+y^{2}}$, $\Delta \theta_{i}=(\theta -\xi_{i})\mathrm{mod}(2\pi )$, θ=arctan2(y,x), −π≤arctan2(y,x)≤π, ω is the angular velocity of the trajectory as it moves around the limit cycle, and $z_{0}$ is used for modeling the baseline wander of the ECG signal. Each of the P, Q, R, S, and T waves of the ECG waveform is modeled with a Gaussian function, and all are located at specific angular positions $\xi_{i}$, and $a_{i}$, $b_{i}$ in Equation (1), and they correspond to the amplitude and width parameters of the Gaussian function.

Then, a simplified dynamic model in its discrete form was used, as proposed by Sameni [15], with the assumption of a small sampling period as follows:

(2) $$\{\begin{array}{c} \theta_{k+1}=(\theta_{k}+\omega \delta )\mathrm{mod}\left(2\pi \right)\\ z_{k+1}=-\sum_{i\in \{Q,P,R,S,T\}}\delta \frac{\alpha_{i}\omega }{b_{i}^{2}}\Delta \theta_{i,k}\exp(-\frac{\Delta \theta_{i,k}^{2}}{2b_{i}^{2}})+z_{k}+\eta_{k} \end{array}$$

where θ is the phase, z is the amplitude of the ECG, and η is a random additive noise.

## 3. Proposed Methodology for FECG Extraction

### 3.1. Modified Abdominal Signal Dynamical Model

It is well understood that the abdominal electrocardiogram (AECG) is a mixture of the MECG, FECG and noise. Thus, we consider the amplitude z and phase θ in Equation (3) of the FECG and the amplitude z and phase θ in Equation (4) of the MECG as state variables so that the new dynamic model can be used to estimate the MECG and FECG simultaneously:

(3) $$\left\{ \begin{array}{l} \theta_{k+1}^{f}=(\theta_{k}^{f}+\omega^{f}\delta )\mathrm{mod}(2\pi )\\ z_{k+1}^{f}=-\sum_{i=1}^{5}\delta \frac{\alpha_{i}^{f}\omega^{f}}{{\left(b_{i}^{f}\right)}^{2}}\Delta \theta_{i,k}^{f}\exp(-\frac{{\left(\Delta \theta_{i,k}^{f}\right)}^{2}}{2{\left(b_{i}^{f}\right)}^{2}})+z_{k}^{f}+\eta_{k}^{f} \end{array}\right.$$

(4) $$\left\{ \begin{array}{l} \theta_{k+1}^{m}=(\theta_{k}^{m}+\omega^{m}\delta )\mathrm{mod}(2\pi )\\ z_{k+1}^{f}=-\sum_{i=1}^{5}\delta \frac{\alpha_{i}^{f}\omega^{f}}{{\left(b_{i}^{f}\right)}^{2}}\Delta \theta_{i,k}^{f}\exp(-\frac{{\left(\Delta \theta_{i,k}^{f}\right)}^{2}}{2{\left(b_{i}^{f}\right)}^{2}})+z_{k}^{f}+\eta_{k}^{f} \end{array}\right.$$

In addition, most previous Bayesian ECG analyses directly use Equation (2) as the state space representation instead of the basic parameters of the Gaussian wave. As a result, occasional morphologic disturbances from random muscle artifacts have considerable effects on the performance of the filter. In these cases, the filter does not have sufficient time to adapt the Gaussian parameters, and a slight phase error of the model can lead to large errors in the Gaussian parameters [16]. The morphology of the ECG is determined by the Gauss parameters $\alpha_{i}$, $b_{i}$, and $\xi_{i}$; therefore, $\alpha_{i}$, $b_{i}$, and $\xi_{i}$ are also considered state variables following the random noise ε as follows:

(5) $$\left\{ \begin{array}{l} \alpha_{k+1}^{f}=\alpha_{k}^{f}+\eta_{\alpha ,k}\\ b_{k+1}^{f}=b_{k}^{f}+\eta_{b,k}\\ \xi_{k+1}^{f}=\xi_{k}^{f}+\eta_{\xi ,k} \end{array}\right.$$

(6) $$\left\{ \begin{array}{l} \alpha_{k+1}^{m}=\alpha_{k}^{m}+\eta_{\alpha ,k}\\ b_{k+1}^{m}=b_{k}^{m}+\eta_{b,k}\\ \xi_{k+1}^{m}=\xi_{k}^{m}+\eta_{\xi ,k} \end{array}\right.$$

where the superscripts f and m in Equations (5) and (6) denote the fetus and mother, respectively. The modified abdominal ECG dynamical model is shown in Equation (7), $x_{k}$ represents state variables, and $\eta_{k}$ represents process noise. The initial values of the dynamical model parameters can be obtained using the automatic off-line parameter selection procedure proposed in [15]:

(7) $$\{\begin{array}{l} x_{k}={\left[\theta_{k}^{f},z_{k}^{f},{\left(\alpha_{k}^{f}\right)}^{T},{\left(b_{k}^{f}\right)}^{T},{\left(\xi_{k}^{f}\right)}^{T},\theta_{k}^{m},z_{k}^{m},{\left(\alpha_{k}^{m}\right)}^{T},{\left(b_{k}^{m}\right)}^{T},{\left(\xi_{k}^{m}\right)}^{T}\right]}^{T}\\ \eta_{k}={\left[\eta_{\theta ,k}^{f},\eta_{z,k}^{f},{\left(\eta_{\alpha ,k}^{f}\right)}^{T},{\left(\eta_{b,k}^{f}\right)}^{T},{\left(\eta_{\xi ,k}^{f}\right)}^{T},\eta_{\theta ,k}^{m},\eta_{z,k}^{m},{\left(\eta_{\alpha ,k}^{m}\right)}^{T},{\left(\eta_{b,k}^{m}\right)}^{T},{\left(\eta_{\xi ,k}^{m}\right)}^{T}\right]}^{T}\\ x_{k+1}=x_{k}+\eta_{k} \end{array}$$

### 3.2. Observation Equation of the Dynamical Model

The phase observation and the noise ECG measurements are related to the measurement vectors described by Sameni [15]. The MECGs can be between five and 10 times greater in intensity than the FECGs, and thus, the extracted FECG signals will be distorted when only the abdominal signal is considered an observation. Thus, in this paper, two auxiliary observation equations are added as observations. These equations are derived from Equation (2), and both are deformation formulas of Equation (2):

(8) $$s_{k}-z_{k-1}^{f}+\sum_{i=1}^{5}\delta \frac{\alpha_{i}^{f}\omega^{f}}{{\left(b_{i}^{f}\right)}^{2}}\Delta \theta_{i,k-1}^{f}\exp(-\frac{{\left(\Delta \theta_{i,k-1}^{f}\right)}^{2}}{2{\left(b_{i}^{f}\right)}^{2}})=z_{k}^{m}+v_{k}^{m}$$

(9) $$s_{k}-z_{k-1}^{m}+\sum_{i=1}^{5}\delta \frac{\alpha_{i}^{m}\omega^{m}}{{\left(b_{i}^{m}\right)}^{2}}\Delta \theta_{i,k-1}^{m}\exp(-\frac{{\left(\Delta \theta_{i,k-1}^{m}\right)}^{2}}{2{\left(b_{i}^{m}\right)}^{2}})=z_{k}^{f}+v_{k}^{f}$$

where Equation (8) is the auxiliary FECG observation equation, and Equation (9) is the auxiliary MECG observation equation. Both equations can be used to estimate the MECG and FECG more accurately. The corresponding observation equations are as follows:

(10) $$\{\begin{array}{c} \varphi_{k}^{f}=\theta_{k}^{f}+u_{k}^{f}\\ s_{k}-z_{k-1}^{m}+\sum_{i=1}^{5}\delta \frac{\alpha_{i}^{m}\omega^{m}}{{\left(b_{i}^{m}\right)}^{2}}\Delta \theta_{i,k-1}^{m}\exp(-\frac{{\left(\Delta \theta_{i,k-1}^{m}\right)}^{2}}{2{\left(b_{i}^{m}\right)}^{2}})=z_{k}^{f}+v_{k}^{f}\\ \varphi_{k}^{m}=\theta_{k}^{m}+u_{k}^{m}\\ s_{k}-z_{k-1}^{f}+\sum_{i=1}^{5}\delta \frac{\alpha_{i}^{f}\omega^{f}}{{\left(b_{i}^{f}\right)}^{2}}\Delta \theta_{i,k-1}^{f}\exp(-\frac{{\left(\Delta \theta_{i,k-1}^{f}\right)}^{2}}{2{\left(b_{i}^{f}\right)}^{2}})=z_{k}^{m}+v_{k}^{m} \end{array}$$

where $u_{k}^{f}$, $v_{k}^{f}$, $u_{k}^{m}$, and $v_{k}^{m}$ are observation noise. The advantage of MPF is that it can approximate non-Gaussian filtering problems; however, to compare it with the Kalman filter, we assumed that the observation noise is Gaussian white noise.

### 3.3. Parallel-Marginalized Particle Filter

The basic idea of a marginalized particle filter (Rao-Blackwellized particle filter) divides the state vector into linear components and nonlinear components [13]. Using the Rao-Blackwell theorem, we marginalized out the linear state variables and estimated them using the Kalman filter, while the particle filter was used to estimate the nonlinear state variables [17]. Thus, the optimal estimation of the state variables was obtained in the linear condition by the Kalman filter, while the dimension of the state variables was reduced in the nonlinear condition by the particle filter. Therefore, the marginalized particle filter algorithm greatly improves the accuracy when using the same number of particles.

In this paper, the par-MPF algorithm is proposed based on the improved AECG dynamic model, which was used to estimate the abdominal signal. Finally, the FECG and MECG can be obtained in parallel. We consider Gaussian amplitude parameters $\alpha_{k}^{f}$, $\alpha_{k}^{m}$ as linear variables and the other parameters as the nonlinear variables [17]. Therefore, the state variable $x_{k}$ can be split into $x_{k}^{L}$ and $x_{k}^{NL}$, which are referred to as linear state and nonlinear variables, respectively:

(11) $$\left\{ \begin{array}{l} x_{k}^{L}={\left[{\left(\alpha_{k}^{f}\right)}^{T},{\left(\alpha_{k}^{m}\right)}^{T}\right]}^{T}\\ x_{k}^{NL}={\left[\theta_{k}^{f},z_{k}^{f},{\left(b_{k}^{f}\right)}^{T},{\left(\xi_{k}^{f}\right)}^{T},\theta_{k}^{m},z_{k}^{m},{\left(b_{k}^{m}\right)}^{T},{\left(\xi_{k}^{m}\right)}^{T}\right]}^{T} \end{array}\right.$$

Using the Bayesian theorem, we can calculate the following:

(12) $$p(x_{k}^{NL},x_{k}^{L}|Y_{k})=p(x_{k}^{L}|x_{k}^{NL},Y_{k})p(x_{k}^{NL},Y_{k})$$

where $p(x_{k}^{L}|x_{k}^{NL},Y_{k})$ is estimated by the Kalman filter, and $p(x_{k}^{NL},Y_{k})$ is estimated by the particle filter.

In summary, the par-MPF algorithm presented in this paper includes the following steps:

*Step 1*: Initialize the M particles:
  (13) $$\left\{ \begin{array}{l} {\left\{x_{0|-1}^{NL,(i)}\right\}}_{i=1}^{M}\sim p(x_{0}^{NL})\\ {\left\{x_{0|-1}^{L,(i)}\right\}}_{i=1}^{M}\sim p(x_{0}^{L}) \end{array}\right.,$$
  and set k=0.
*Step 2*: Calculate the importance:
  weight:
  (14) $$w_{k}^{\left(i\right)}=p(Y_{k}|x_{k|k-1}^{NL,(i)})\;i=1,2,\cdots ,M;$$
  normalization:
  (15) $$w_{k}^{\left(i\right)}=w_{k}^{\left(i\right)}/\sum_{i=1}^{M}w_{k}^{\left(i\right)}.$$
*Step 3*: Resample the M particles:
  (16) $$p(x_{k|k}^{NL,(i)}=x_{k|k-1}^{NL,(j)})=w_{k}^{\left(j\right)}$$
*Step 4*: Update the Kalman filter measurement:
  (17) $$\left\{ \begin{array}{l} {\tilde{\alpha }}_{k|k}^{f/m,(i)}={\tilde{\alpha }}_{k|k-1}^{f/m,(i)}\\ P_{k|k}^{f/m}=P_{k|k-1}^{f/m} \end{array}\right.,$$
  where the superscript f/m means that there are two cases, i.e., the fetal ECG and the maternal ECG estimates.
*Step 5*: Update the particle filter time:
  (18) $$p(x_{k+1|k}^{f/mNL,(i)}|x_{k|k}^{f/mNL,(i)})=N(\mu_{k}^{f/m},\Sigma_{k}^{f/m})$$
  where:
  (19) $$\left\{ \begin{array}{l} \mu_{k}^{f/m}={\left[\theta_{k}^{f/m,(i)}+w^{f/m}\delta ,-g^{T}(x_{k}^{f/mNL,(i)})\alpha_{k}^{f/m,(i)}+z_{k}^{f/m,(i)},b_{k}^{f/m,(i)},\xi_{k}^{f/m,(i)}\right]}^{T}\\ \Sigma_{k}^{f/m}=diag(\sigma_{\theta^{f/m}}^{2},\sigma_{z^{f/m}}^{2},\sigma_{b^{f/m}}^{2},\sigma_{\xi^{f/m}}^{2})\\ g(x_{k}^{NL})=[g(x_{k,1}^{NL}),g(x_{k,2}^{NL}),g(x_{k,3}^{NL}),g(x_{k,4}^{NL}),g(x_{k,5}^{NL})]\\ g(x_{k,j}^{NL})=\delta \frac{\alpha_{j,k}\omega }{b_{j,k}^{2}}\Delta \theta_{j,k}\exp(-\frac{\Delta \theta_{j,k}^{2}}{2b_{j,k}^{2}}) \end{array}\right.$$
*Step 6*: Update the Kalman filter time:
  (20) $${\tilde{\alpha }}_{k+1|k}^{f/m,(i)}={\tilde{\alpha }}_{k|k}^{f/m,(i)}+L_{k}^{f/m}({\tilde{z}}_{k+1|k}^{f/m,(i)}-z_{k}^{f/m,(i)}-g^{T}(x_{k+1|k}^{f/mNL,(i)}){\tilde{\alpha }}_{k|k}^{f/m,(i)})$$
  where:
  (21) $$\left\{ \begin{array}{l} F_{k}^{f/m}=g^{T}({\tilde{x}}_{k+1|k}^{f/mNL,(i)})P_{k|k}^{f/m,(i)}g({\tilde{x}}_{k+1|k}^{f/mNL,(i)})+Q_{k}^{f/mNL}\\ L_{k}^{f/m}=P_{k|k}^{f/m,(i)}g({\tilde{x}}_{k+1|k}^{f/mNL,(i)}){\left(F_{k}^{f/m}\right)}^{-1} \end{array}\right.,$$
  P is the process noise covariance matrix, and Q is the observation noise covariance matrix.
*Step 7*:
  (22) k=k+1,
  and iterate from step 2.

## 4. Performance Index of the FECG Extraction

### 4.1. Performance Index of the Simulated Data

For the simulated data, the percentage root mean-square difference (PRD) and the signal-to-noise ratio enhancement ($SNR_{enh}$) were used to estimate the performance of filtering algorithms. The percentage root mean-square difference is defined as follows [18]:

(23) $$PRD=\frac{\sum_{n}{\left(f(n)-\hat{f}(n)\right)}^{2}}{\sum_{n}{\left(f(n)\right)}^{2}}$$

where f(n) is the original FECG signal, $\hat{f}(n)$ denotes the extracted FECG, and PRD is the most frequently used interference measurement tool that enumerates the error between the original signal and the reconstructed signal. The nearer the PRD is to zero, the more it indicates the similarity between the two signals and, hence, indicates the signal quality.

The signal-to-noise ratio enhancement is defined as follows [19]:

(24) $$SNR_{enh}=10\log\frac{\sum_{n}{\left(a(n)-f(n)\right)}^{2}}{\sum_{n}{\left(f(n)-\hat{f}(n)\right)}^{2}}$$

where a(n) is the abdominal signal, f(n) is the original FECG signal, and $\hat{f}(n)$ denotes the extracted FECG. $SNR_{enh}$ denotes the difference between the SNR before and after filtering. A larger $SNR_{enh}$ indicates a better performance of the algorithm.

### 4.2. Performance Index of the Clinical Data

For the clinical data, the signal-to-noise ratio is based on an eigenvalue analysis and a cross correlation can be used to estimate the performance of filtering algorithms. Jagannath D.J. et al. [18], used SNR, including $SNR_{\mathrm{svd}}$, the quotient between the first singular value (fetus signal energy) and the sum of the rest of the singular values (noise energy), and $SNR_{cor}$, as an indicator of the goodness of the extraction method. For a square matrix, similarly, the eigenvalue was used to represent signal energy instead. Both of the SNR values can be calculated by the following steps [6]:

*Step 1*: The extracted FECG is divided into N pieces crossing to the R peak, and each piece includes M samples and one QRS complex.
*Step 2*: All of the pieces are stored in columns of an M by N matrix U, and the vectors u(k) are the zero mean and are normalized to the unit length; that is,
  (25) $$u^{T}(k)u(k)=1.$$
*Step 3*: A signal-to-noise ratio based on eigenvalues can be calculated as:
  (26) $$SNR_{eig}=\frac{\lambda_{\max}}{N-\lambda_{\max}}$$
  where λ contains the eigenvalues of U.
*Step 4*: A signal-to-noise ratio based on the cross-correlation coefficients can be calculated as
  (27) $$SNR_{cor}=\frac{\eta }{1-\eta }$$
  where
  (28) $$\eta =\frac{2}{M(M-1)}\sum_{i=0}^{M-2}\sum_{k=i+1}^{M-1}f{\left(i\right)}^{T}f(k),$$
  and f is the FECG.

## 5. Results and Analysis

To verify the feasibility and reliability of the proposed algorithm, both simulated and clinical data were used to study the performance of the proposed method, and other methods were tested with the same signals, including EKS, EKF and the Bayesian adaptive neuro fuzzy inference system [18]. The main ideas of these algorithms are shown in Table 1.

### 5.1. FECG Extraction on the Simulated Data

#### 5.1.1. Simulated Data without Noise

**Figure 1:**
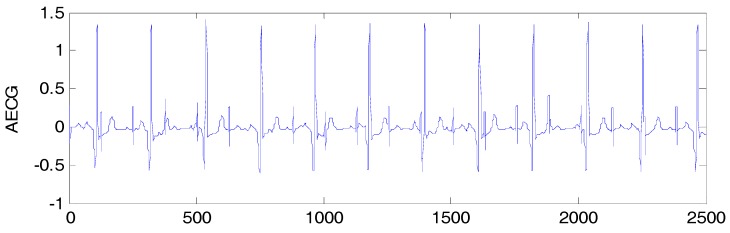
Simulated data.

**Figure 2:**
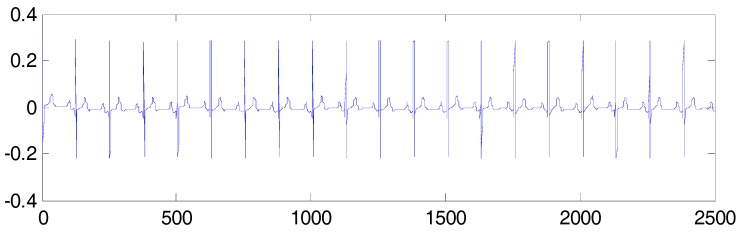
Simulated FECG.

An artificial FECG-MECG mixture simulator, as proposed by Behar [20], was used for creating the simulated data, where the MECG was simulated with a beat rate of 70 bpm, and the FECG was simulated with a beat rate of 120 bpm. The sampling frequency was set to 250 Hz, and the acquisition time was 10 s. The parameter $SNR_{fm}=\frac{\sum_{n}fecg{\left(n\right)}^{2}}{\sum_{n}mecg{\left(n\right)}^{2}}=-10\mathrm{dB}$, and a synthetic AECG signal is shown in Figure 1. The simulated FECG signal is shown in Figure 2.

**Figure 3:**
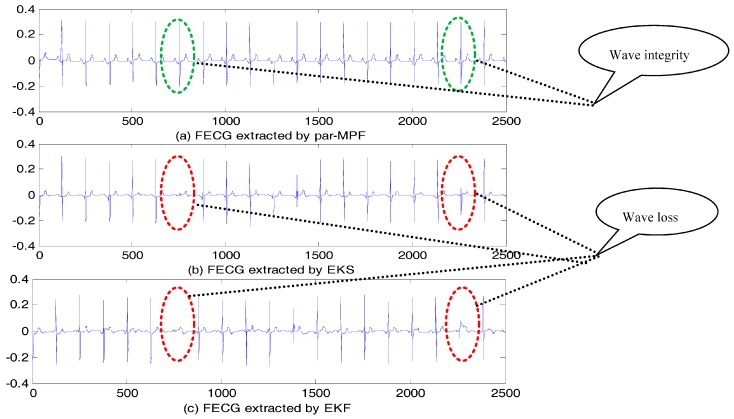
FECG estimation: (**a**) FECG extracted by par-MPF; (**b**) FECG extracted by EKS; (**c**) FECG extracted by EKF.

The FECG signals extracted are shown in Figure 3. It is clear that all three algorithms correctly separate the mixed signal, and from the extracted FECG, we can see that unlike par-MPF, it does not fail when the MECG and FECG overlap. This performance was particularly noted for the samples between 700 and 800 and between 2200 and 2300 in Figure 3, in which some parts of the FECG signal deteriorated during the MECG extraction by the EKS and EKF methods. In contrast, the proposed par-MPF jointly models the FECG and MECG, resulting in a better estimate of the FECG. The reason for this is that some parts of the FECG signal deteriorated during the MECG extraction by the EKS and EKF methods. In contrast, the proposed method obtains the MECG and FECG at the same time and results in a better estimate of the FECG.

**Figure 4:**
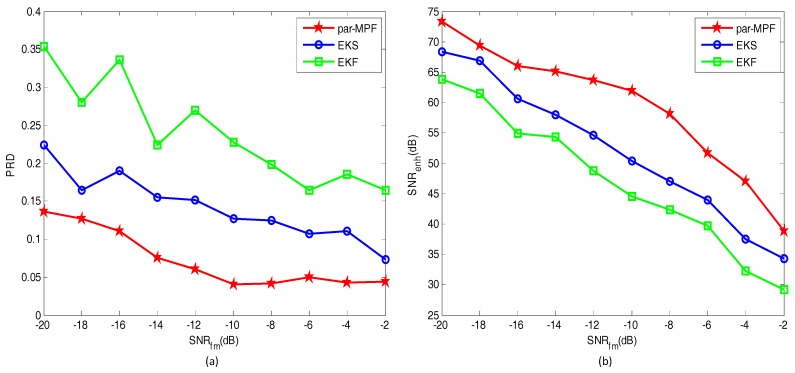
Comparison of performance indices: (**a**) PRD; (**b**) $SNR_{enh}$.

Another 10 FECG datasets, in which $SNR_{fm}=-2,-4\cdots ,-18,-20\mathrm{dB}$, were used to further verify the proposed algorithm. Three filtering algorithms were imposed on these signals to split the MECG and FECG, and the PRD and $SNR_{enh}$ of the extracted FECG are shown in Figure 4a,b, respectively.

As shown in Figure 4, when the $SNR_{fm}$ increases, the PRD value of the par-MPF is smaller than the EKS and EKF, while the $SNR_{enh}$ is higher than the EKS and EKF. Overall, the proposed method has a stable performance; thus, the algorithm can be applied to the simulated data with noise.

#### 5.1.2. Simulated Data with Noise

**Figure 5:**
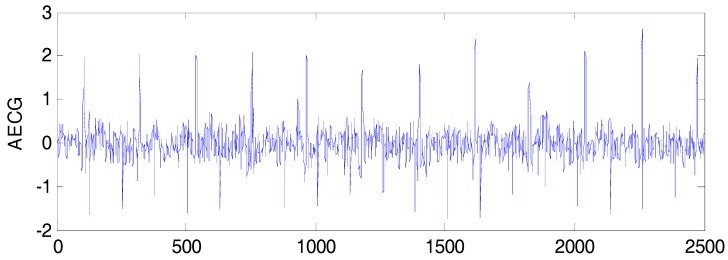
Simulated data with noise.

The artificial FECG-MECG mixture simulator was used again to generate the AECG with a motion artifact, as shown in Figure 5, in which $SNR_{fm}=-10\;\mathrm{dB}$, and $SNR_{mn}=6\;\mathrm{dB}$, keeping the other conditions the same:

(29) $$SNR_{mn}=\frac{\sum_{n}[fecg{\left(n\right)}^{2}+mecg{\left(n\right)}^{2}]}{\sum_{n}noise{\left(n\right)}^{2}}$$

**Figure 6:**
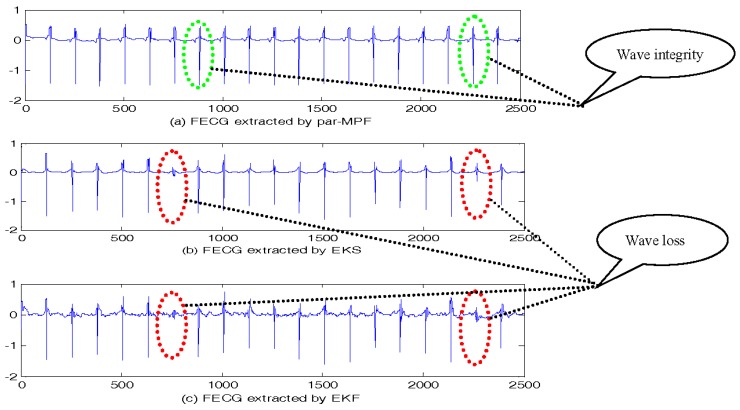
FECG estimation: (**a**) FECG extracted by par-MPF; (**b**) FECG extracted by EKS; (**c**) FECG extracted by EKF.

To verify the feasibility of the proposed algorithm, the AECG in Figure 5 was processed by the three filtering methods. The extracted FECG is shown in Figure 6. The EKS and EKF also fail when the MECG and FECG overlap, while the par-MPF does not fail. This experiment shows that the algorithm is feasible for the FECG separation of the clinical signal.

**Figure 7:**
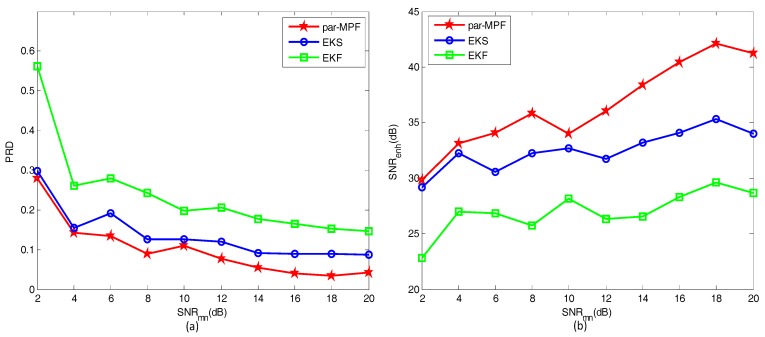
Comparison of performance indices: (**a**) PRD; (**b**) $SNR_{enh}$.

We changed the values of $SNR_{mn}$, after which the PRD and $SNR_{enh}$ were calculated to evaluate the performance of these filtering methods. The results of the PRD and $SNR_{enh}$ calculations are shown in Figure 7a,b, respectively, when $SNR_{mn}=2,4,\cdots ,18,20\;\mathrm{dB}$.

Figure 7 clearly demonstrates that when $SNR_{mn}$ increases, the PRD of the par-MPF is smaller than the EKS and EKF, while the $SNR_{enh}$ is higher than the EKS and EKF. In summary, when the AECG is mixed with different noise, the proposed algorithm shows a perfect filtering performance, and the extracted FECG is high quality. Therefore, the application of the filtering algorithm to the clinical AECG should be considered.

### 5.2. FECG Extraction on the Different Database.

#### 5.2.1. Database for the Identification of Systems

**Figure 8:**
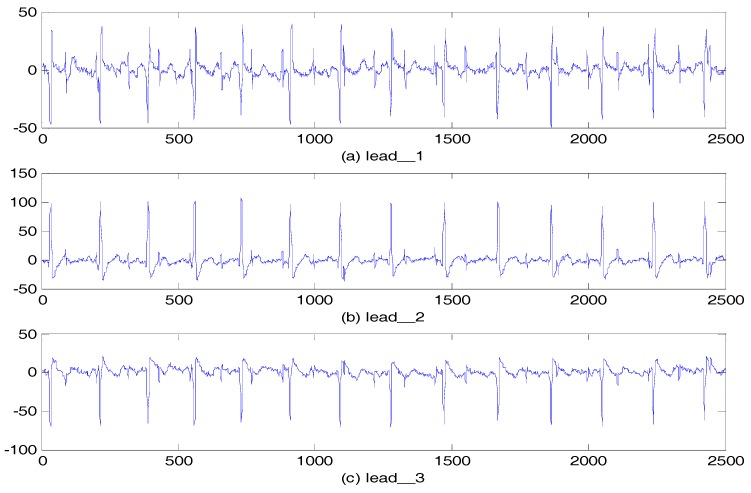

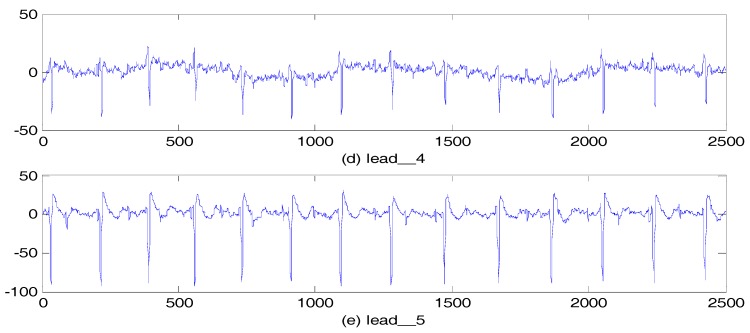
AECG: (**a**) Lead_1; (**b**) Lead_2; (**c**) Lead_3; (**d**) Lead_4; (**e**) Lead_5.

The clinical data are from the Database for the Identification of Systems (DaISy), which is a well-known ECG measured from a pregnant woman. A total of eight channels are available, which are sampled at 250 Hz with a duration of 10 s [21]. Five abdominal signals are shown in Figure 8. The FECG components in lead_1 were the most obvious, while the lead_4 signal had so much noise that the FECG components could hardly be seen.

**Figure 9:**
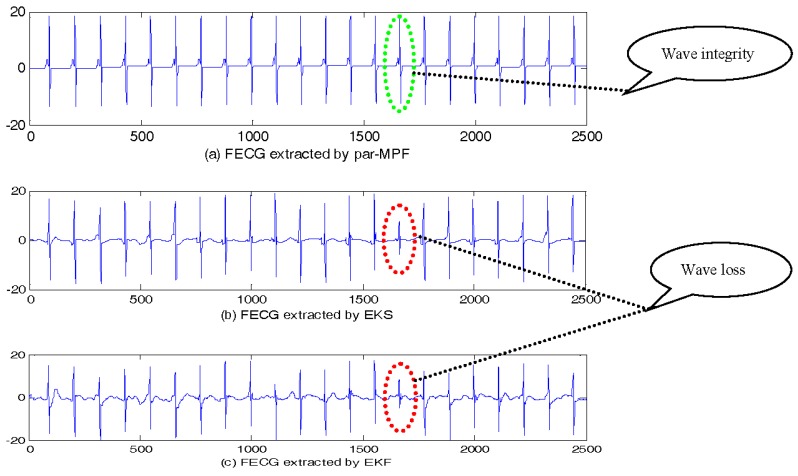

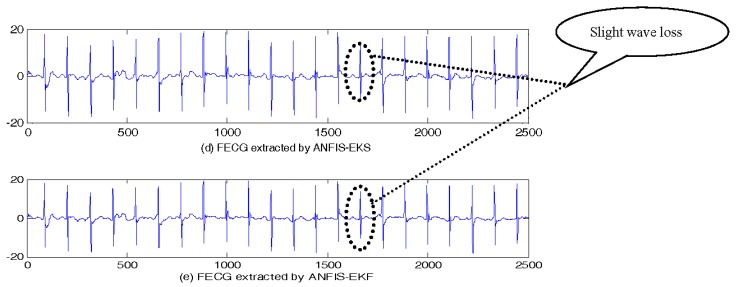
FECG estimation: (**a**) FECG extracted by par-MPF; (**b**) FECG extracted by EKS; (**c**) FECG extracted by EKF; (**d**) FECG extracted by ANFIS-EKS; (**e**) FECG extracted by ANFIS-EKF.

We ran the proposed algorithm and the other three algorithms. Figure 9 presents the FECG extracted using the lead_1. The proposed method resulted in a superior estimation of the FECG compared to the other methods. When the MECG and FECG completely overlap in samples between 1600 and 1700, there is a small effect on the ANFIS-EKS and ANFIS-EKF outputs, while the FECGs suffer a severe loss when extracted by the EKS and EKF. As described above, there are two steps in extracting the FECG signal with the EKS and EKF algorithms. The FECG is obtained from the residual signal, which will reduce the QRS wave of the FECG component, while the par-MPF algorithm is based on the improved AECG model and extracts the MCG and FECG in a parallel manner. Thus, the FECG is estimated more accurately, and the performance of the algorithm is greatly improved.

**Figure 10:**
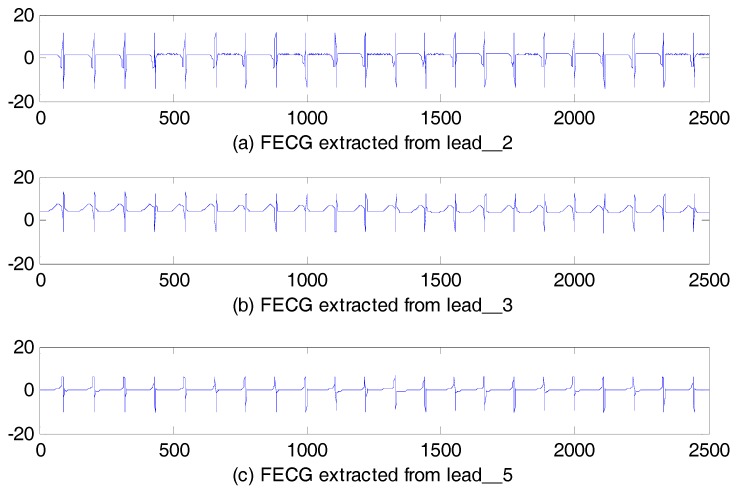
FECG estimation: (**a**) FECG extracted from lead_2; (**b**) FECG extracted from lead_3; (**c**) FECG extracted from lead_5.

The FECG signals obtained using the par-MPF from three other abdominal signals are shown in Figure 10. To further illustrate the advantages of our algorithm, another experiment was conducted using another AECG without lead_4. The $SNR_{eig}$ and $SNR_{cor}$ are calculated in Table 2.

From the experimental results, we can see that the signal-to-noise ratio of our algorithm is higher than that of the other four algorithms; furthermore, the difference between the signal-to-noise ratio of the par-MPF is small. It can be concluded that the performance of the algorithm is better than that of the other Bayesian algorithms.

#### 5.2.2. Abdominal and Direct Fetal ECG Database

**Figure 11:**
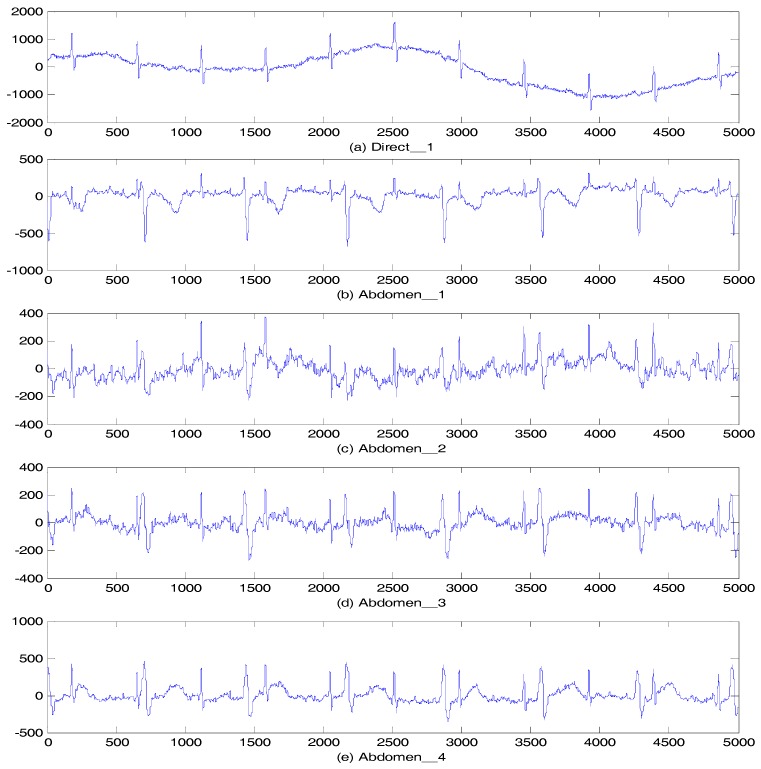
Signal r01: (**a**) Direct_1; (**b**) Abdomen_1; (**c**) Abdomen_2; (**d**) Abdomen_3; (**e**) Abdomen_4.

The clinical data used in this section are from the Abdominal and Direct Fetal ECG Database. The five-minute multichannel fetal ECG recordings, with cardiologist-verified annotations of all fetal heartbeats, were from five women in labor at the Medical University of Silesia, Poland. Each record includes four signals from the maternal abdomen and a simultaneously recorded reference direct fetal ECG from the fetal scalp; all of the signals were sampled at 1 KHz [22,23]. The first 5000 points were used to plot the ECG waveform as shown in Figure 11, where (a) is the fetal scalp ECG signal, and (b) to (e) are the abdominal signals.

**Figure 12:**
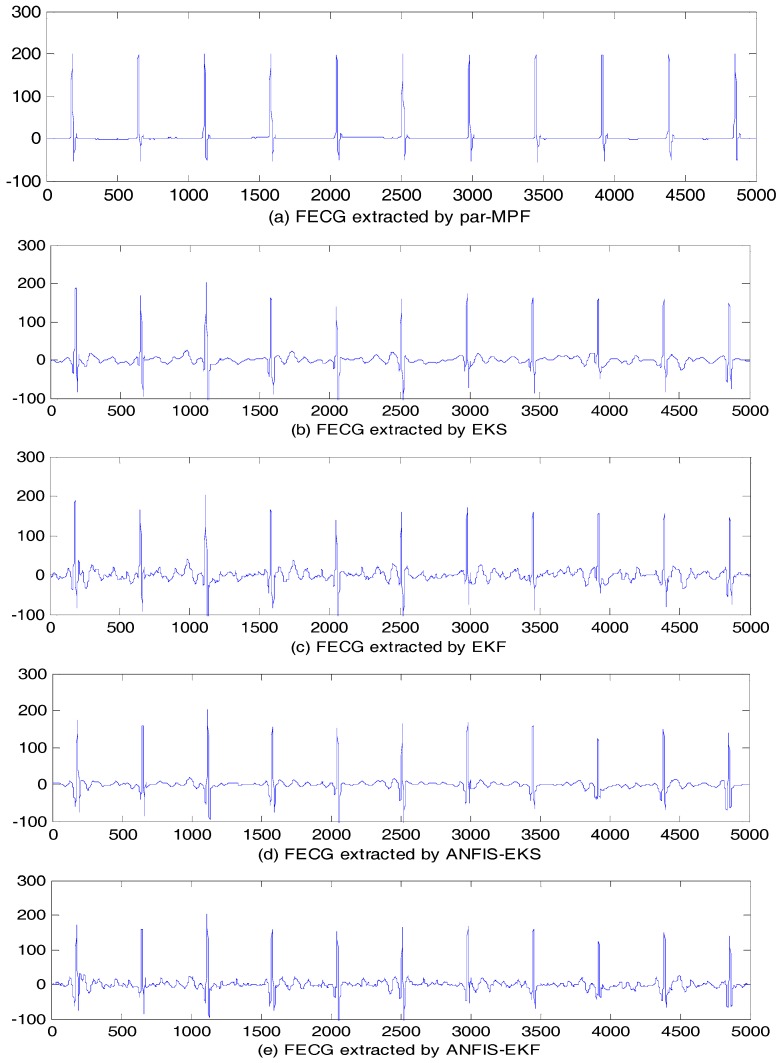
FECG estimation: (**a**) FECG extracted by par-MPF; (**b**) FECG extracted by EKS; (**c**) FECG extracted by EKF; (**d**) FECG extracted by ANFIS-EKS; (**e**) FECG extracted by ANFIS-EKF.

The result of the filtering algorithms using the signal Abdomen_1 are shown in Figure 12. These fives algorithms adapted to the different shapes of the AECG, which included the interference of noise. However, the performance of the EKF was the worst among these algorithms, and the FECG was mixed with more noise. The results of the FECG extraction using EKS, ANFIS-EKS, and ANFIS-EKF had relatively low noise. In contrast, the FECG extraction using the par-MPF had the best quality.

**Figure 13:**
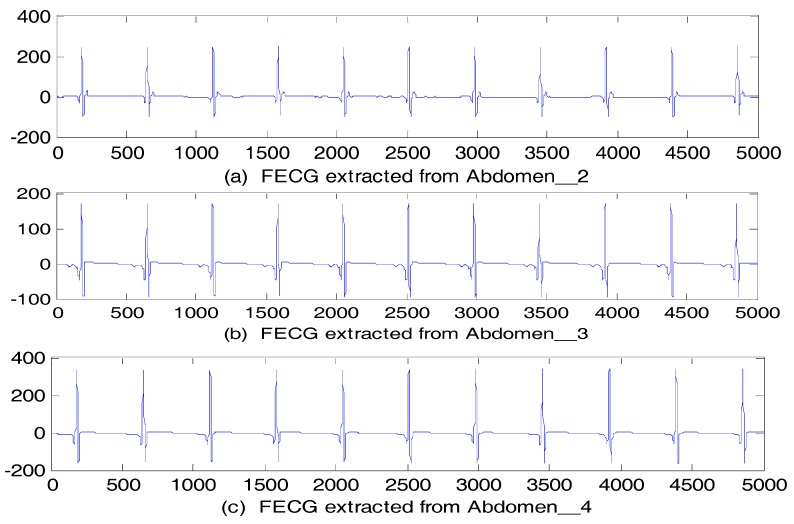
FECG estimation: (**a**) FECG extracted from Abdomen_2; (**b**) FECG extracted from Abdomen_3; (**c**) FECG extracted from Abdomen_4.

The FECG signals obtained using the par-MPF by the three other abdominal signals of r01 are shown in Figure 13; the algorithm performance was evaluated by calculating the $SNR_{eig}$ and $SNR_{cor}$ using these three signals of r01, and the results are shown in Table 3. All of the algorithms extracted the FECG with different signal qualities; however, the proposed algorithm not only extracted the FECG without noise, but the quality of the signal was finer, and thus, it is helpful for the follow-up extraction of the R wave and FHRV analysis.

**Figure 14:**
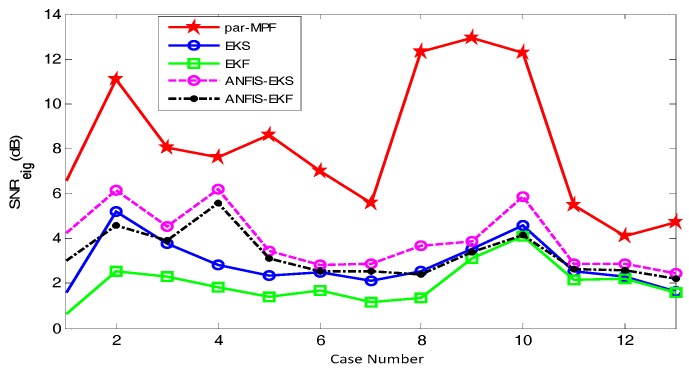
Comparison of the $SNR_{eig}$.

**Figure 15:**
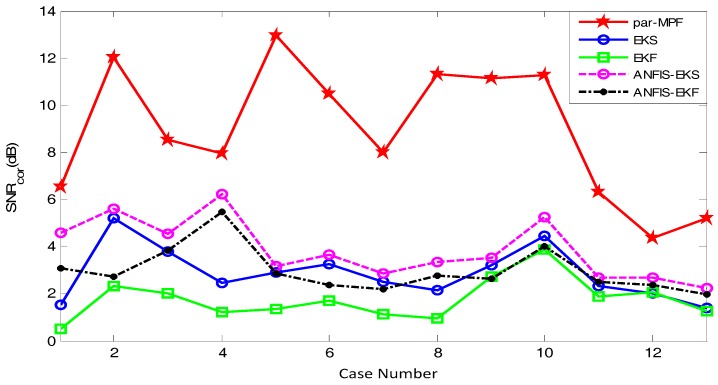
Comparison of the $SNR_{cor}$.

Finally, the signals r04, r07, r08 and r10 in the Abdominal and Direct Fetal ECG Database were processed using the above filtering algorithm [24,25,26]. Because the signals of Abdomen_2 of r07, Abdomen_1 of r08 and Abdomen_2 of r10 were poor, they were excluded from the experiments. The comparison of the $SNR_{eig}$ and $SNR_{cor}$ among the five algorithms is shown in Figure 14 and Figure 15, respectively.

In Figure 14 and Figure 15, the X-axis and Y-axis indicate the number of the signal used in these experiments and the SNR, respectively. From Figure 14 and Figure 15, we can clearly see that for all signals tested, the SNR of the proposed method is higher than that for KS, EKF, ANFIS-EKS and ANFIS-EKF. The higher SNR here means a better extraction performance. Figure 14 and Figure 15 show that the performance indices of the proposed method are obviously higher than those of the other algorithms, and no matter how the abdominal ECG changes, the algorithm maintains a good performance. The EKF achieved the lowest SNR and showed the poorest performance. The results indicate that the algorithm in this paper effectively extracted a high-quality FECG from different pregnant women. Therefore, this algorithm has great prospects for clinical application.

## 6. Conclusions

A Bayesian filtering framework for extracting the FECG from a single-channel mixture of the abdominal signal MECG and FECG, including noise, is proposed in this paper. A modified abdominal signal dynamical model is proposed to improve the filtering accuracy. The FECG and MECG are obtained at the same time, substantially reducing the running time of the algorithm. In addition, simulated and clinical data were used to verify the performance of the filter. All the results show that the proposed method successfully extracted the FECG in many scenarios, and the extracted FECG had a high quality.

Finally, the proposed method was compared with some other Bayesian filter algorithms, such as the EKS, EKF and the Bayesian adaptive neuro fuzzy inference system. The proposed method provided a superior estimation of the FECGs compared with the other methods, and when the MECG and FECG completely overlapped, there was little effect on the ANFIS-EKS and ANFIS-EKF. However, the FECGs suffered a severe loss when extracted by the EKS and EKF methods. Moreover, the performance indices of the proposed method were obviously higher than those of the other algorithms. Taken together, these experiments reveal that the strength of the proposed method is the best with respect to signal extraction. The proposed par-MPF algorithm has been verified to obtain a high signal-to-noise ratio FECG signal from a single-channel abdominal signal. However, whether the algorithm is suited for the extraction of twin FECGs is an issue for further study.

## Figures and Tables

**Table 1:** Filtering algorithms used in the experiments.

Filtering Algorithm	Main Idea
par-MPF	Marginalized particle filter
EKS [10]	Extended Kalman filter + Smooth
EKF [11]	Extended Kalman filter
ANFIS-EKS [12]	EKS + Adaptive neuro fuzzy inference system
ANFIS-EKF [12]	EKF + Adaptive neuro fuzzy inference system

**Table 2:** The SNR based on the eigenvalues and the cross-correlation coefficients.

Methods	Lead 1		Lead 2		Lead 3		Lead 4	
	$SNR_{eig}$	$SNR_{cor}$	$SNR_{eig}$	$SNR_{cor}$	$SNR_{eig}$	$SNR_{cor}$	$SNR_{eig}$	$SNR_{cor}$
par-MPF	2.0864	1.6820	2.0849	1.7120	2.4119	2.1926	2.1033	1.7037
EKS [10]	1.7497	1.5677	1.8250	1.3714	1.3903	0.7797	1.1414	0.9533
EKF [11]	1.2245	1.1506	1.2367	0.8392	1.3590	0.7465	0.6258	0.4130
ANFIS-EKS [12]	1.8334	1.6152	1.8709	1.4889	1.4452	1.4917	1.1837	0.9856
ANFIS-EKF [12]	1.5097	1.3160	1.5491	1.2557	1.3767	0.9247	0.8465	0.6551

**Table 3:** SNR based on the eigenvalues and the cross-correlation coefficients of r01.

Method	Abdomen-1		Abdomen-2		Abdomen-3		Abdomen-4	
	$SNR_{eig}$	$SNR_{cor}$	$SNR_{eig}$	$SNR_{cor}$	$SNR_{eig}$	$SNR_{cor}$	$SNR_{eig}$	$SNR_{cor}$
par-MPF	12.8898	14.7332	12.1464	13.9624	11.4695	13.2144	13.352	15.3952
EKS [10]	3.5025	3.6133	3.8609	4.1144	5.0389	5.1286	6.7488	6.9799
EKF [11]	2.4222	2.3902	2.236	2.2596	3.5868	3.2583	4.2651	3.8168
ANFIS-EKS [12]	4.2285	4.5879	4.1438	4.6256	5.5427	5.5549	7.2039	7.2104
ANFIS-EKF [12]	3.0071	3.0873	2.5573	2.7417	3.9021	3.8444	5.5771	5.4508

## References

[1] Lukosevicius M., Marozas V. (2014). Noninvasive fetal QRS detection using an echo state network and dynamic programming. Physiol. Meas..

[2] Ayat M., Assaleh K., Al-Nashash H. Extracting fetal ECG from a single maternal abdominal record. Proceedings of the IEEE 8th GCC Conference and Exhibition (GCCCE).

[3] Puthusserypady S. (2007). Extraction of fetal electrocardiogram using H∞ adaptive algorithms. Med. Biol. Eng. Comput..

[4] Zhang W., Liu H.X., Cheng J.C. (2012). Adaptive filtering in phase space for foetal electrocardiogram estimation from an abdominal electrocardiogram signal and a thoracic electrocardiogram signal. IET Signal Process..

[5] Zheng W., Wei X.Y., Zhong J.J., Liu H.X. (2013). Noninvasive fetal ECG estiomation using adaptive comb filter. Comput. Methods Progr. Biomed..

[6] Wu S., Shen Y., Zhou Z., Lin L., Zeng Y., Gao X. (2013). Research of fetal ECG extraction using wavelet analysis and adaptive filtering. Comput. Biol. Med..

[7] Elmansouri K., Latif R., Maoulainine F. Efficient fetal heart rate extraction using undecimated wavelet transform. Proceedings of the 2nd World Conference on Complex Systems (WCCS).

[8] Desai K.D., Sankhe M.S. A real-time fetal ECG feature extraction using multiscale discrete wavelet transform. Proceedings of the 5th International Conference on Biomedical Engineering and Informatics (BMEI).

[9] Campolo M., Labate D., Foresta F.L., Morabito F.C. ECG-derived respiratory signal using Empirical Mode Decomposition. Proceedings of the IEEE International Workshop on Medical Measurements and Applications.

[10] Niknazar M., Rivet B., Jutten C. (2013). Fetal ECG extraction by extended state kalman filtering based on single-channel recordings. IEEE Trans. Biomed. Eng..

[11] Zeng X., Zhou X., Li G., Liu Q. Robust adaptive fetal heart rate estimation for single-channel abdominal ECG recording. Proceedings of the 5th International Conference on Biomedical Engineering and Informatics (BMEI).

[12] Panigrahy D., Sahu P.K. (2015). Extraction of fetal electrocardiogram (ECG) by extended state Kalman filtering and adaptive neuro-fuzzy inference system (ANFIS) based on single channel abdominal recording. Sadhana.

[13] Schon T., Gustafsson F., Nordlund P. (2005). Marginalized particle filters for mixed linear/nonlinear state-space models. IEEE Trans. Signal Process..

[14] McSharry P.E., Clifford G.D., Tarassenko L., Smith L.A. (2003). A dynamical model for generating synthetic electrocardiogram signals. IEEE Trans. Biomed. Eng..

[15] Sameni R., Shamsollahi M.B., Jutten C., Clifford G.D. (2007). A nonlinear Bayesian filtering framework for ECG denoising. IEEE Trans. Biomed. Eng..

[16] Li G., Zeng X., Lin J., Zhou X. Genetic particle filtering for denoising of ECG corrupted by muscle artifacts. Proceedings of the 8th International Conference on Natural Computation.

[17] Lin C., Bugallo M., Mailhes C., Tourneret J.Y. ECG denoising using a dynamical model and a marginalized particle filter. Proceedings of the Conference Record of the 45th Asilomar Conference on Signals, Systems and Computers (ASILOMAR).

[18] Jagannath D.J., Selvakumar A.I. (2015). Superior foetal electrocardiogram signal elicitation using a novel artificial intelligent Bayesian methodology. Appl. Soft Comput..

[19] Martinez M., Calpe J., Soria E., Guerrero J.F. Methods to evaluate the performance of fetal electrocardiogram extraction algorithms. Proceedings of the Computers in Cardiology 2001.

[20] Behar J., Andreotti F., Zaunseder S., Li Q., Oster J., Clifford G.D. (2014). An ECG simulator for generating maternal-foetal activity mixtures on abdominal ECG recordings. Physiol. Meas..

[21] L.LathauwerDaISy: Database for the Identification of Systems. http://www.esat.kuleuven.ac.be./sista/daisy.

[22] Jezewski J., Matonia A., Kupka T., Roj D., Czabanski R. (2012). Determination of fetal heart rate from abdominal signals: Evaluation of beat-to-beat accuracy in relation to the direct fetal electrocardiogram. Biomed. Tech..

[23] Abdominal and Direct Foetal ECG Database. https://www.physionet.org/physiobank/database/adfecgdb.

[24] Karvounis E.C., Tsipouras M.G., Fotiadis D.I. (2009). Detection of Fetal Heart Rate through 3-D Phase Space Analysis from Multivariate Abdominal Recordings. IEEE Trans. Biomed. Eng..

[25] Castillo E., Morales D.P., Botella G., Garcia A., Parrilla L., Palma A.J. (2013). Efficient wavelet-based ECG processing for single-lead FHR extraction. Digit. Signal Process..

[26] Castillo E., Morales D.P., Garcia A., Martinez-Marti F., Parrilla L., Palma A.J. (2013). Noise Suppression in ECG Signals through Efficient One-Step Wavelet Processing Techniques. J. Appl. Math..

